# Crystal structure and Hirshfeld surface analysis of (2*E*)-1-phenyl-3-(1*H*-pyrrol-2-yl)propen-1-one

**DOI:** 10.1107/S2056989024000495

**Published:** 2024-01-26

**Authors:** Ayten S. Safarova, Ali N. Khalilov, Mehmet Akkurt, Abel M. Maharramov, Ajaya Bhattarai, Farid N. Naghiyev, İbrahim G. Mamedov

**Affiliations:** a Department of Chemistry, Baku State University, Z. Khalilov str. 23, AZ1148, Baku, Azerbaijan; b ‘Composite Materials’ Scientific Research Center, Azerbaijan State Economic University (UNEC), H. Aliyev str. 135, AZ1063, Baku, Azerbaijan; c Department of Physics, Faculty of Sciences, Erciyes University, 38039 Kayseri, Türkiye; d Department of Chemistry, M.M.A.M.C. (Tribhuvan University) Biratnagar, Nepal; University of Neuchâtel, Switzerland

**Keywords:** crystal structure, 1*H*-pyrrole ring, hydrogen bond, chalcone, Hirshfeld surface analysis

## Abstract

The title com­pound, C_13_H_11_NO, adopts an *E* configuration about the C=C double bond. The pyrrole ring is inclined to the phenyl ring at an angle of 44.94 (8)°. In the crystal, mol­ecules are linked by N—H⋯O hydrogen bonds, forming ribbons parallel to (020) in zigzag *C*(7) chains along the *a* axis. These ribbons are connected *via* C—H⋯π inter­actions, forming a three-dimensional network. No significant π–π inter­actions are observed.

## Abstract 2

In the title crystal, mol­ecules are linked by N—H⋯O hydrogen bonds, forming ribbons parallel to (020) in zigzag *C*(7) chains along the *a* axis. These ribbons are connected *via* C—H⋯π inter­actions, forming a three-dimensional network.

## 1. Chemical context

The chemistry of carbo- and heterocyclic aromatic com­pounds is the most important branch of organic chemistry (Khalilov *et al.*, 2022 ▸; Akkurt *et al.*, 2023 ▸). Synthetic organic chemistry is growing tremendously, with recently developed aromatic systems that are designed for various research and commercial purposes (Maharramov *et al.*, 2021 ▸, 2022 ▸; Erenler *et al.*, 2022 ▸). Nowadays, five- and six-membered heterocyclic systems find broad applications in different branches of chemistry, as well as coordination chemistry (Gurbanov *et al.*, 2021 ▸; Mahmoudi *et al.*, 2021 ▸), drug design and development (Donmez & Turkyılmaz, 2022 ▸; Askerova, 2022 ▸), and materials science (Velásquez *et al.*, 2019 ▸; Afkhami *et al.*, 2019 ▸). The pyrrole motif is the most widespread five-membered heteroaromatic ring system in nitro­gen heterocycles (Mahmoudi *et al.*, 2017 ▸). It is an essential structural motif present in many natural tetra­pyrrole scaffolds of heme and related cofactors (chloro­phyll a, heme b, vitamin B12 and factor 430), and other bioactive mol­ecules, like porphobilinogen, nargenicin, prodigiosin, *etc.* (Walsh *et al.*, 2006 ▸; Sobhi & Faisal, 2023 ▸). Chalcones incorporating N-heterocyclic, especially pyrrole, scaffolds with various biological and pharmacological activities, such as anti­oxidant, anti­bacterial, anti­fungal, anti­leishmanial, anti­cancer, anti­tubercular, anti­malarial and other properties, have been reviewed recently (Mezgebe *et al.*, 2023 ▸). In addition, the incorporation of diverse pharmacophore groups in a pyrrole scaffold has led to the development of more desired com­pounds, such as elopiprazole, lorpiprazole, isamoltane, obatoclax, *etc.* (Bhardwaj *et al.*, 2015 ▸; Atalay *et al.*, 2022 ▸). Thus, in the framework of our studies in heterocyclic chemistry (Naghiyev *et al.*, 2020 ▸, 2021 ▸, 2022 ▸), we report herein the crystal structure and Hirshfeld surface analysis of the title com­pound, (2*E*)-1-phenyl-3-(1*H*-pyrrol-2-yl)propen-1-one. 
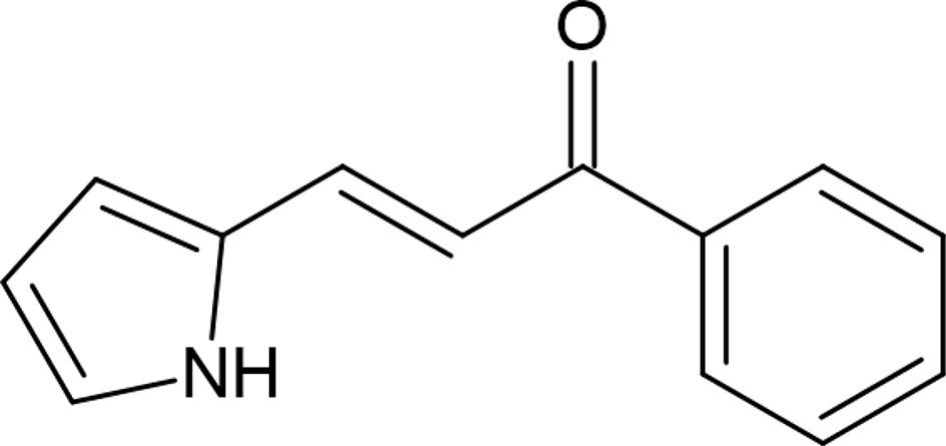


## 2. Structural commentary

**Figure 1:**
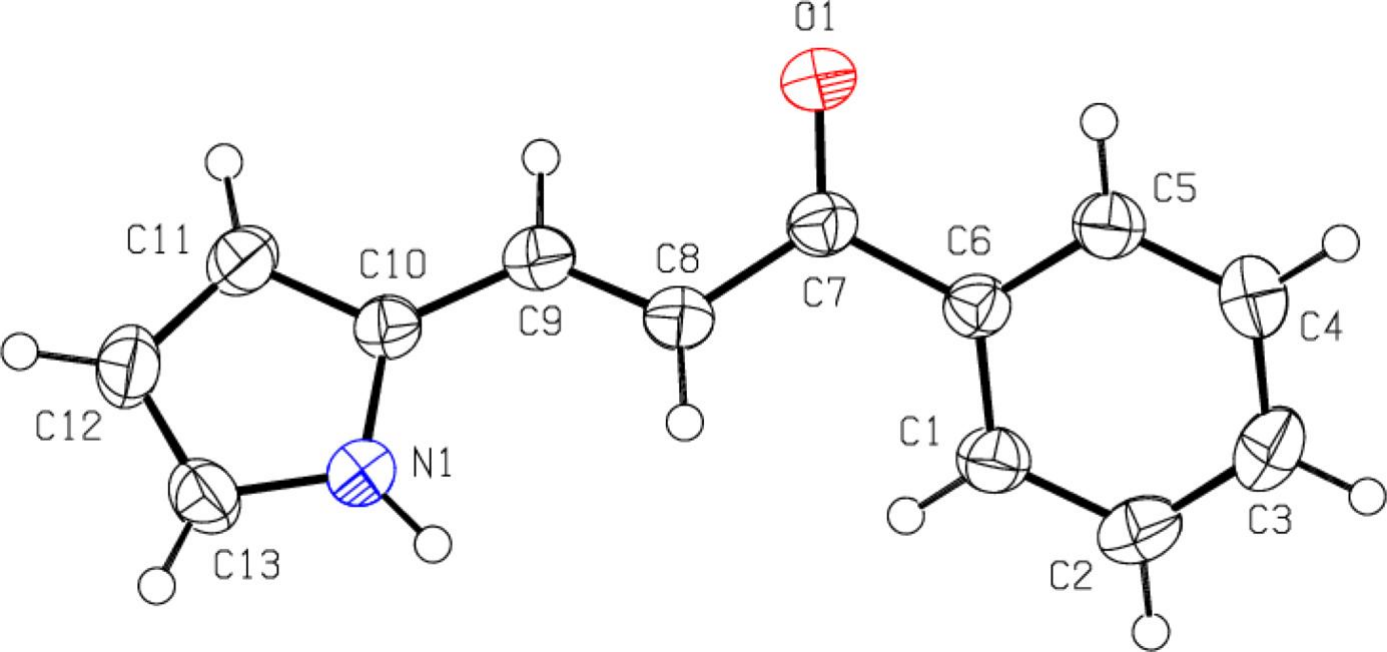
The mol­ecular structure of the title com­pound, showing the atom labelling and displacement ellipsoids drawn at the 50% probability level.

The title com­pound (Fig. 1 ▸) shows an *E* configuration about the C=C double bond. The pyrrole ring (atoms N1/C10–C13) is inclined to the phenyl ring (C1–C6) by 44.94 (8)°, the torsion angles being C5—C6—C7—C8 = −156.04 (13)°, C5—C6—C7—O1 = 22.6 (2)°, C6—C7—C8—C9 = −163.76 (13)°, C7—C8—C9—C10 = −172.34 (13)°, O1—C7—C8—C9 = 17.6 (2)° and C8—C9—C10—C11 = 173.37 (14)°. The geometrical parameters of the the title com­pound are in agreement with those reported for similar com­pounds; see the *Database survey* section.

## 3. Supra­molecular features and Hirshfeld surface analysis

**Figure 2:**
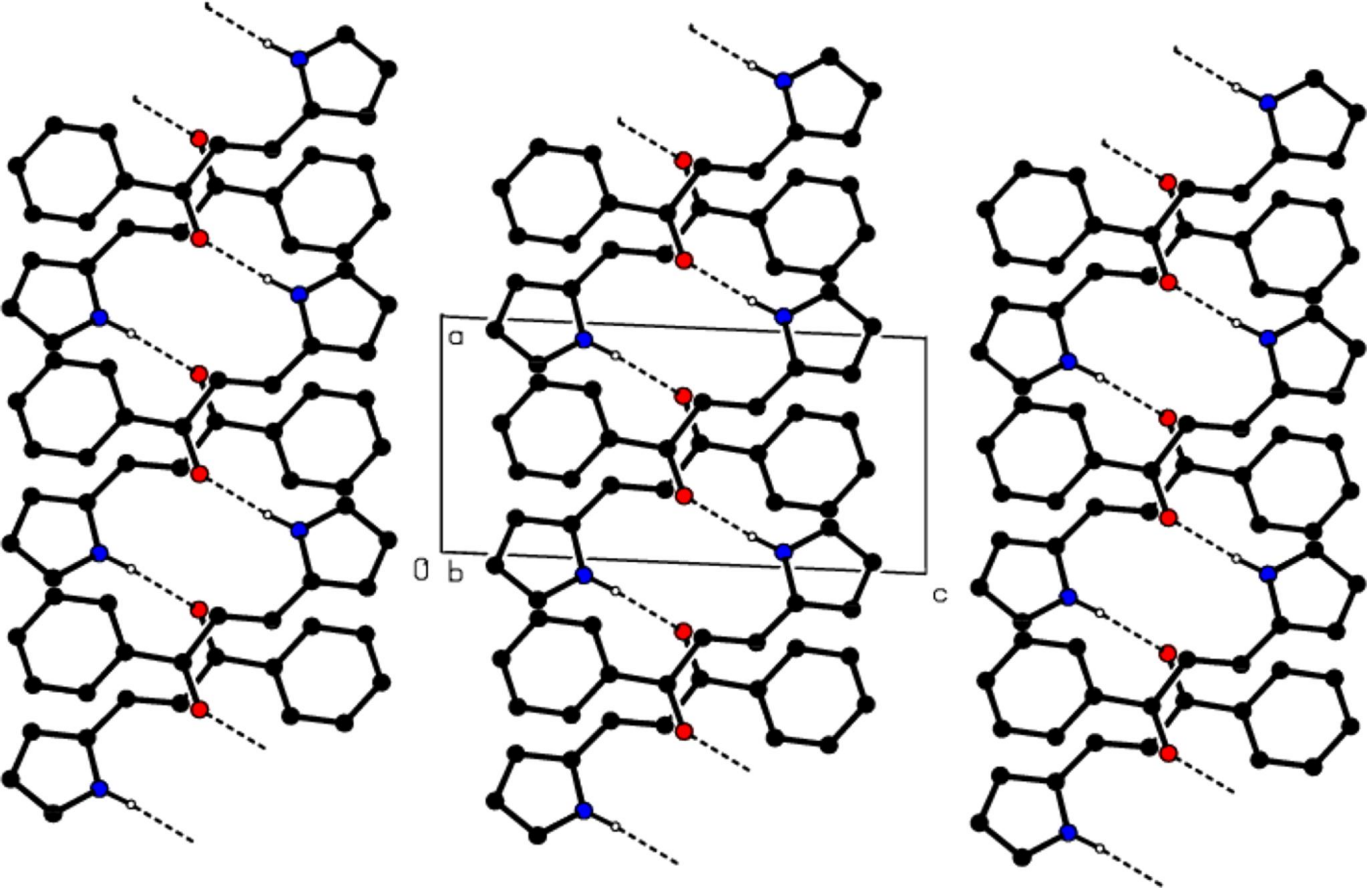
View of the N—H⋯O hydrogen bonds of the title com­pound down the *b* axis.

**Figure 3:**
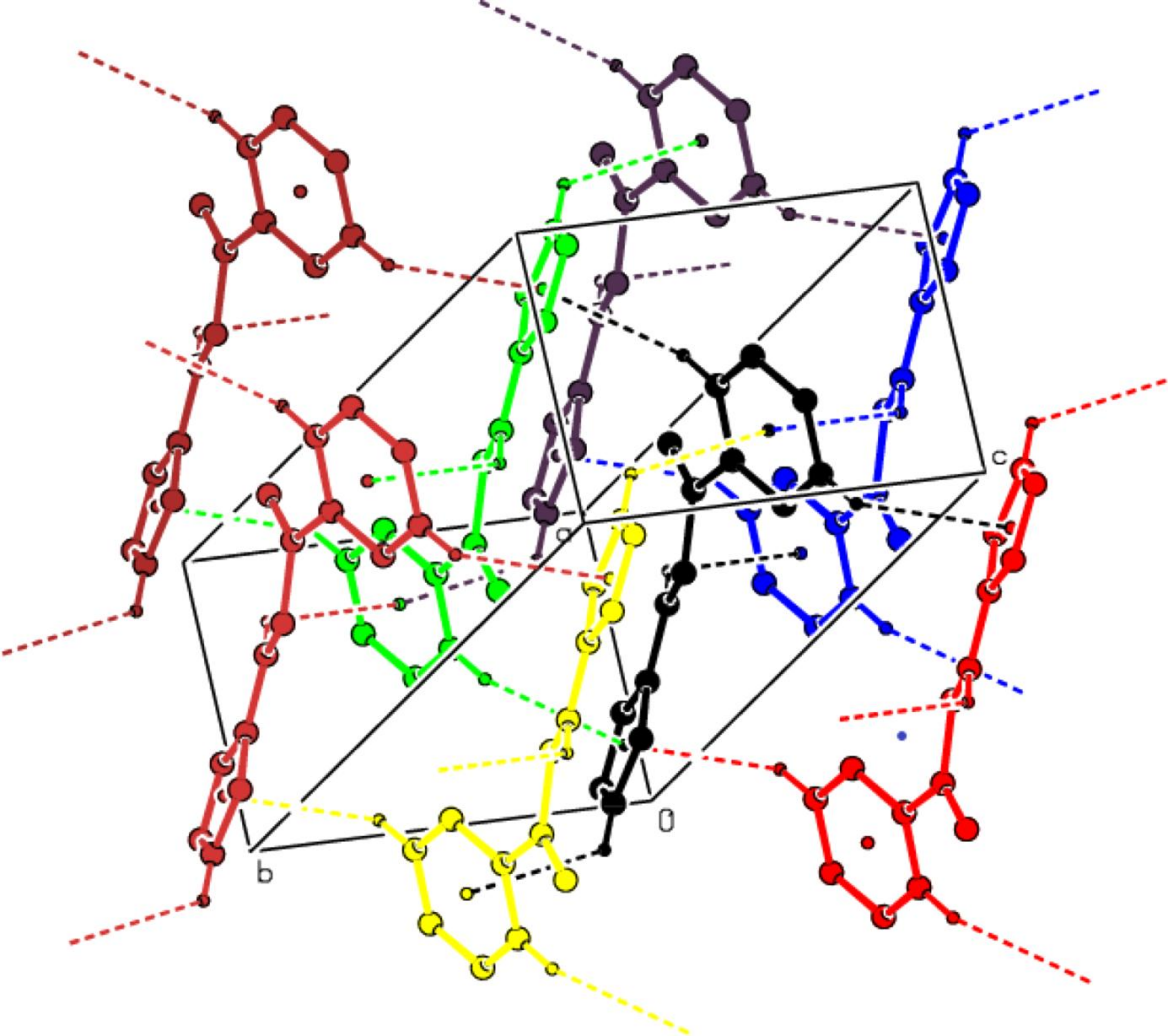
View of the C—H⋯π inter­actions of the title com­pound in the unit cell.

In the crystal, mol­ecules are linked by N—H⋯O hydrogen bonds, forming ribbons parallel to (020) in zigzag *C*(7) chains along the *a* axis (Bernstein *et al.*, 1995 ▸; Table 1 ▸ and Fig. 2 ▸). These ribbons are connected *via* C—H⋯π inter­actions, forming a three-dimensional network (Table 1 ▸ and Fig. 3 ▸). No significant π–π inter­actions are observed.

**Figure 4:**
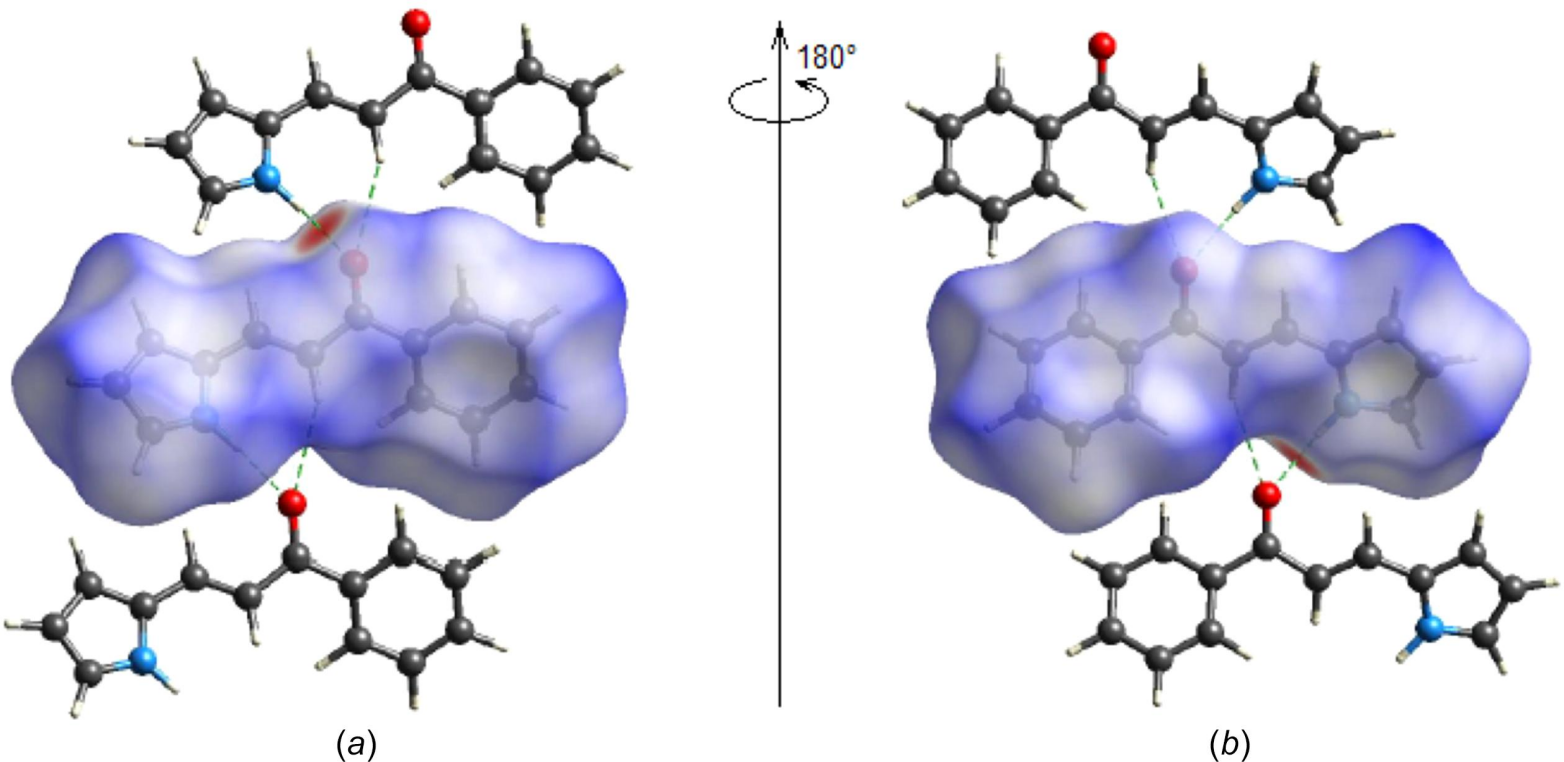
(*a*) Front and (*b*) back sides of the three-dimensional Hirshfeld surface of the title com­pound mapped over *d*_norm_, with a fixed colour scale of −0.4746 to +1.2616 a.u.

The Hirshfeld surfaces of the title mol­ecule and the two-dimensional fingerprints were com­puted with *CrystalExplorer17.5* (Spackman *et al.*, 2021 ▸). The *d*_norm_ mappings for the title com­pound were performed in the range from −0.4746 (red) to +1.2616 (blue) a.u. On the *d*_norm_ surfaces, bright-red spots indicate the locations of the N—H⋯O inter­actions [Table 1 ▸ and Figs. 4 ▸(*a*) and 4(*b*)].

**Figure 5:**
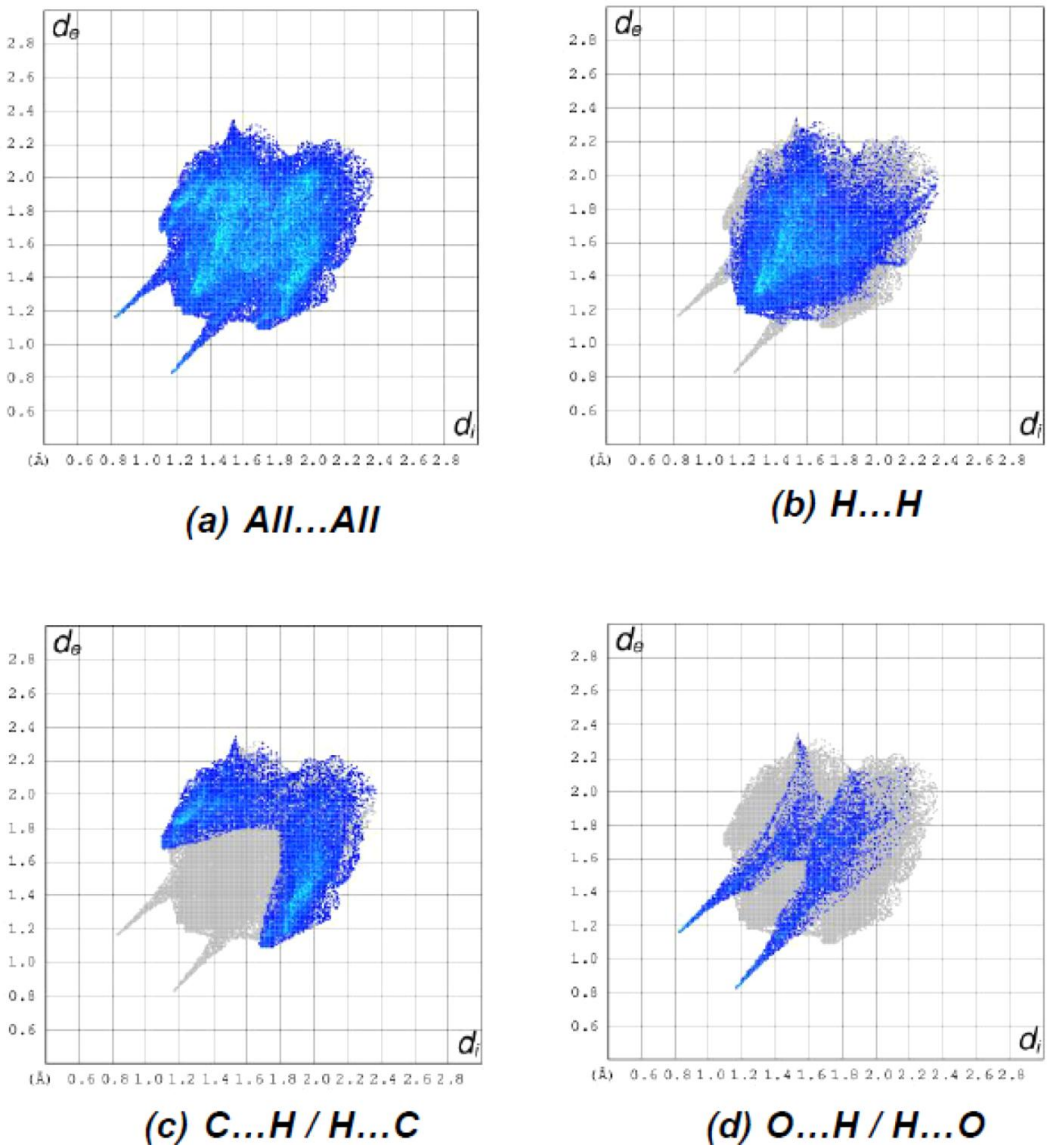
The two-dimensional fingerprint plots of the title com­pound, showing (*a*) all inter­actions, and delineated into (*b*) H⋯H, (*c*) C⋯H/H⋯C and (*d*) O⋯H/H⋯O inter­actions.

The fingerprint plots (Fig. 5 ▸) reveal that while H⋯H inter­actions [Fig. 5 ▸(*b*); 48.4%] make the largest contributions to the surface contacts (Table 1 ▸), C⋯H/H⋯C [Fig. 5 ▸(*c*); 31.7%] and O⋯H/H⋯O [Fig. 5 ▸(*d*); 11.3%] contacts are also important. Other less notable inter­actions are C⋯C (3.7%), N⋯H/H⋯N (3.1%) and O⋯C/C⋯O (1.8%).

## 4. Database survey

A search of the Cambridge Structural Database (CSD, Version 5.43, last update November 2022; Groom *et al.*, 2016 ▸) for the ‘(2*E*)-1-phenyl-3-(1*H*-pyrrol-2-yl)prop-2-en-1-one’ skeleton of the title com­pound yielded two hits, namely, 1-(3-chloro­phen­yl)-3-(3-fur­yl)prop-2-en-1-one (CSD refcode NUQFOW; Zingales *et al.*, 2015 ▸) and (*E*)-3-(2-fur­yl)-1-phenyl­prop-2-en-1-one (NOTCUW01; Vázquez-Vuelvas *et al.*, 2015 ▸). When the positions of the pyrrole and phenyl rings are switched, additional hits are found, namely, 1-(2,4-di­methyl­furan-3-yl)-3-phenyl­prop-2-en-1-one (MISXUL; Khalilov *et al.*, 2023 ▸), 1-(3-fur­yl)-3-[3-(tri­fluoro­meth­yl)phen­yl]prop-2-en-1-one (KUDNAA; Bákowicz *et al.*, 2015 ▸) and (2*E*)-3-(4-chloro­phen­yl)-1-(1*H*-pyrrol-2-yl)prop-2-en-1-one (XIYYOU; Bukhari *et al.*, 2008 ▸).

In the crystal of NUQFOW, mol­ecules stack along the *a* axis; however, there are no significant inter­molecular inter­actions present. In the crystal of NOTCUW01, mol­ecules are connected by weak C—H⋯O hydrogen bonds and C—H⋯π inter­actions, forming ribbons extending along the *c* axis. In the crystal of MISXUL, pairs of mol­ecules are linked by C—H⋯O hydrogen bonds, forming dimers with 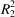 (14) ring motifs. The mol­ecules are connected *via* C—H⋯π inter­actions, forming a three-dimensional network. No π–π inter­actions are observed. In KUDNAA, mol­ecules are linked by inter­molecular C—H⋯O inter­actions, forming zigzag chains with *C*(5) motifs along the *b* axis. In addition, mol­ecules are connected by face-to-face π–π stacking inter­actions [centroid–centroid distances = 3.926 (3) and 3.925 (2) Å] between the opposing benzene and furan rings of the mol­ecules along the *c* axis. In XIYYOU, inter­molecular N—H⋯O hydrogen bonds link the mol­ecules into centrosymmetric 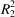 (10) dimers. There are C—H⋯π inter­actions between the benzene and pyrrole rings and a benzene C—H group. A weak π–π inter­action between the pyrrole rings [centroid–centroid distance = 3.8515 (11) Å] further stabilizes the structure. There is also a π-inter­action between the pyrrole ring and the carbonyl group, with an O⋯π distance of 3.4825 (18) Å.

## 5. Synthesis and crystallization

The title com­pound was synthesized according to a recently reported procedure (Li *et al.*, 2022 ▸), and colourless crystals were obtained upon recrystallization from an ethanol/water (3:1 *v*/*v*) solution.

## 6. Refinement

Crystal data, data collection and structure refinement details are summarized in Table 2 ▸. The C-bound H atoms were placed in calculated positions (0.93 Å) and refined as riding atoms with *U*_iso_(H) = 1.2*U*_eq_(C). The N-bound H atom was located in a difference map and refined with *U*_iso_(H) = 1.2*U*_eq_(N). Three reflections (001, 010 and 020) were omitted in the final cycles of refinement.

## Supplementary Material

Crystal structure: contains datablock(s) global. DOI: 10.1107/S2056989024000495/tx2080sup1.cif

Supporting information file. DOI: 10.1107/S2056989024000495/tx2080globalsup2.cml

CCDC reference: 2321201

Additional supporting information:  crystallographic information; 3D view; checkCIF report

## Figures and Tables

**Table 1:** Hydrogen-bond geometry (Å, °) *Cg*1 and *Cg*2 are the centroids of the phenyl (C1–C6) and 1*H*-pyrrole (N1/C10–C13) rings, respectively.

*D*—H⋯*A*	*D*—H	H⋯*A*	*D*⋯*A*	*D*—H⋯*A*
N1—H1*N*⋯O1^i^	0.872 (17)	2.119 (17)	2.9561 (18)	160.7 (14)
C2—H2⋯*Cg*1^ii^	0.93	2.82	3.450 (2)	126
C5—H5⋯*Cg*1^iii^	0.93	2.91	3.5233 (19)	124
C9—H9⋯*Cg*2^iv^	0.93	3.00	3.6207 (18)	126
C13—H13⋯*Cg*2^v^	0.93	2.95	3.593 (2)	127

Symmetry codes: (i) 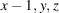 ; (ii) 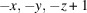 ; (iii) 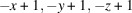 ; (iv) 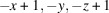 ; (v) 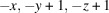 .

**Table 2:** Experimental details

Crystal data	
Chemical formula	C_13_H_11_NO
*M*_r_	197.23
Crystal system, space group	Triclinic, *P* 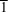
Temperature (K)	293
*a*, *b*, *c* (Å)	5.7855 (16), 7.3347 (19), 12.424 (3)
α, β, γ (°)	106.519 (8), 91.912 (9), 92.326 (9)
*V* (Å^3^)	504.5 (2)
*Z*	2
Radiation type	Mo *K*α
μ (mm^−1^)	0.08
Crystal size (mm)	0.12 × 0.11 × 0.10

Data collection	
Diffractometer	Bruker APEXII CCD
Absorption correction	Multi-scan (*SADABS*; Krause *et al.*, 2015 ▸)
*T*_min_, *T*_max_	0.688, 0.746
No. of measured, independent and observed [*I* > 2σ(*I*)] reflections	18072, 2535, 1908
*R*_int_	0.042
(sin θ/λ)_max_ (Å^−1^)	0.673

Refinement	
*R*[*F* ^2^ > 2σ(*F* ^2^)], *wR*(*F* ^2^), *S*	0.049, 0.108, 1.06
No. of reflections	2535
No. of parameters	139
H-atom treatment	H atoms treated by a mixture of independent and constrained refinement
Δρ_max_, Δρ_min_ (e Å^−3^)	0.18, −0.16

Computer programs: *APEX2* and *SAINT* (Bruker, 2007 ▸), *SHELXT* (Sheldrick, 2015*a* ▸), *SHELXL* (Sheldrick, 2015*b* ▸), *ORTEP-3 for Windows* (Farrugia, 2012 ▸) and *PLATON* (Spek, 2020 ▸).

## supplementary crystallographic information

### Crystal data

**Table:**

C_13_H_11_NO	*Z* = 2
*M**_r_* = 197.23	*F*(000) = 208
Triclinic, *P*1	*D*_x_ = 1.298 Mg m^−^^3^
*a* = 5.7855 (16) Å	Mo *K*α radiation, λ = 0.71073 Å
*b* = 7.3347 (19) Å	Cell parameters from 5813 reflections
*c* = 12.424 (3) Å	θ = 2.9–27.4°
α = 106.519 (8)°	µ = 0.08 mm^−^^1^
β = 91.912 (9)°	*T* = 293 K
γ = 92.326 (9)°	Block, colorless
*V* = 504.5 (2) Å^3^	0.12 × 0.11 × 0.10 mm

### Data collection

**Table:**

Bruker APEXII CCD diffractometer	1908 reflections with *I* > 2σ(*I*)
φ and ω scans	*R*_int_ = 0.042
Absorption correction: multi-scan (*SADABS*; Krause *et al.*, 2015)	θ_max_ = 28.6°, θ_min_ = 2.9°
*T*_min_ = 0.688, *T*_max_ = 0.746	*h* = −7→7
18072 measured reflections	*k* = −9→9
2535 independent reflections	*l* = −16→16

### Refinement

**Table:**

Refinement on *F*^2^	0 restraints
Least-squares matrix: full	Hydrogen site location: mixed
*R*[*F*^2^ > 2σ(*F*^2^)] = 0.049	H atoms treated by a mixture of independent and constrained refinement
*wR*(*F*^2^) = 0.108	*w* = 1/[σ^2^(*F*_o_^2^) + (0.0348*P*)^2^ + 0.1277*P*] where *P* = (*F*_o_^2^ + 2*F*_c_^2^)/3
*S* = 1.06	(Δ/σ)_max_ < 0.001
2535 reflections	Δρ_max_ = 0.18 e Å^−^^3^
139 parameters	Δρ_min_ = −0.15 e Å^−^^3^

### Special details

**Table:**

Geometry. All esds (except the esd in the dihedral angle between two l.s. planes) are estimated using the full covariance matrix. The cell esds are taken into account individually in the estimation of esds in distances, angles and torsion angles; correlations between esds in cell parameters are only used when they are defined by crystal symmetry. An approximate (isotropic) treatment of cell esds is used for estimating esds involving l.s. planes.

### Fractional atomic coordinates and isotropic or equivalent isotropic displacement parameters (Å^2^)

**Table:**

	*x*	*y*	*z*	*U*_iso_*/*U*_eq_	
C1	0.2761 (2)	0.1954 (2)	0.68844 (12)	0.0384 (3)	
H1	0.152580	0.148144	0.636826	0.046*	
C2	0.2561 (3)	0.1996 (2)	0.79934 (13)	0.0454 (4)	
H2	0.120202	0.152611	0.822105	0.055*	
C3	0.4365 (3)	0.2729 (2)	0.87637 (13)	0.0482 (4)	
H3	0.421898	0.276227	0.951211	0.058*	
C4	0.6386 (3)	0.3416 (2)	0.84307 (13)	0.0455 (4)	
H4	0.759276	0.393419	0.895615	0.055*	
C5	0.6619 (2)	0.3335 (2)	0.73212 (12)	0.0378 (3)	
H5	0.800320	0.376468	0.709453	0.045*	
C6	0.4805 (2)	0.26175 (18)	0.65370 (11)	0.0324 (3)	
C7	0.5128 (2)	0.25283 (19)	0.53401 (11)	0.0354 (3)	
C8	0.3085 (2)	0.2513 (2)	0.46122 (12)	0.0383 (3)	
H8	0.168759	0.287979	0.493566	0.046*	
C9	0.3176 (2)	0.19850 (19)	0.34947 (11)	0.0357 (3)	
H9	0.455504	0.148284	0.321134	0.043*	
C10	0.1436 (2)	0.20911 (19)	0.26803 (11)	0.0335 (3)	
C11	0.1590 (3)	0.1715 (2)	0.15357 (12)	0.0407 (4)	
H11	0.283975	0.120383	0.112231	0.049*	
C12	−0.0454 (3)	0.2235 (2)	0.11041 (13)	0.0457 (4)	
H12	−0.081206	0.214416	0.035462	0.055*	
C13	−0.1829 (3)	0.2901 (2)	0.19837 (13)	0.0436 (4)	
H13	−0.330437	0.334068	0.193755	0.052*	
N1	−0.0693 (2)	0.28171 (17)	0.29363 (10)	0.0386 (3)	
H1N	−0.130 (3)	0.302 (2)	0.3590 (14)	0.046*	
O1	0.70997 (17)	0.25046 (16)	0.49928 (8)	0.0496 (3)	

### Atomic displacement parameters (Å^2^)

**Table:**

	*U*^11^	*U*^22^	*U*^33^	*U*^12^	*U*^13^	*U*^23^
C1	0.0329 (7)	0.0410 (8)	0.0402 (8)	−0.0001 (6)	0.0026 (6)	0.0099 (6)
C2	0.0437 (9)	0.0482 (9)	0.0484 (9)	0.0010 (7)	0.0145 (7)	0.0190 (7)
C3	0.0613 (10)	0.0526 (9)	0.0349 (8)	0.0068 (8)	0.0081 (7)	0.0181 (7)
C4	0.0467 (9)	0.0493 (9)	0.0391 (9)	0.0005 (7)	−0.0068 (7)	0.0115 (7)
C5	0.0338 (7)	0.0404 (8)	0.0402 (8)	0.0016 (6)	0.0029 (6)	0.0128 (6)
C6	0.0320 (7)	0.0324 (7)	0.0331 (7)	0.0059 (5)	0.0044 (6)	0.0089 (5)
C7	0.0335 (7)	0.0379 (7)	0.0349 (7)	0.0053 (6)	0.0059 (6)	0.0096 (6)
C8	0.0315 (7)	0.0474 (8)	0.0371 (8)	0.0078 (6)	0.0052 (6)	0.0127 (6)
C9	0.0328 (7)	0.0373 (7)	0.0380 (8)	0.0013 (6)	0.0052 (6)	0.0120 (6)
C10	0.0320 (7)	0.0354 (7)	0.0334 (7)	−0.0009 (5)	0.0025 (6)	0.0109 (6)
C11	0.0412 (8)	0.0464 (8)	0.0344 (8)	−0.0019 (6)	0.0062 (6)	0.0115 (6)
C12	0.0521 (10)	0.0540 (9)	0.0333 (8)	−0.0052 (7)	−0.0036 (7)	0.0179 (7)
C13	0.0364 (8)	0.0502 (9)	0.0479 (9)	0.0019 (7)	−0.0054 (7)	0.0207 (7)
N1	0.0360 (7)	0.0472 (7)	0.0338 (7)	0.0031 (5)	0.0048 (5)	0.0129 (5)
O1	0.0330 (6)	0.0771 (8)	0.0403 (6)	0.0057 (5)	0.0088 (5)	0.0181 (5)

### Geometric parameters (Å, º)

**Table:**

C1—C2	1.378 (2)	C8—C9	1.3346 (19)
C1—C6	1.3886 (19)	C8—H8	0.9300
C1—H1	0.9300	C9—C10	1.4237 (19)
C2—C3	1.375 (2)	C9—H9	0.9300
C2—H2	0.9300	C10—N1	1.3728 (18)
C3—C4	1.377 (2)	C10—C11	1.3761 (19)
C3—H3	0.9300	C11—C12	1.394 (2)
C4—C5	1.374 (2)	C11—H11	0.9300
C4—H4	0.9300	C12—C13	1.361 (2)
C5—C6	1.3867 (19)	C12—H12	0.9300
C5—H5	0.9300	C13—N1	1.3526 (18)
C6—C7	1.4882 (19)	C13—H13	0.9300
C7—O1	1.2318 (16)	N1—H1N	0.871 (16)
C7—C8	1.462 (2)		
			
C2—C1—C6	120.07 (14)	C9—C8—C7	121.56 (13)
C2—C1—H1	120.0	C9—C8—H8	119.2
C6—C1—H1	120.0	C7—C8—H8	119.2
C3—C2—C1	120.16 (14)	C8—C9—C10	128.38 (13)
C3—C2—H2	119.9	C8—C9—H9	115.8
C1—C2—H2	119.9	C10—C9—H9	115.8
C2—C3—C4	120.20 (14)	N1—C10—C11	106.72 (12)
C2—C3—H3	119.9	N1—C10—C9	124.28 (12)
C4—C3—H3	119.9	C11—C10—C9	128.65 (13)
C5—C4—C3	119.93 (14)	C10—C11—C12	108.08 (13)
C5—C4—H4	120.0	C10—C11—H11	126.0
C3—C4—H4	120.0	C12—C11—H11	126.0
C4—C5—C6	120.48 (13)	C13—C12—C11	107.23 (13)
C4—C5—H5	119.8	C13—C12—H12	126.4
C6—C5—H5	119.8	C11—C12—H12	126.4
C5—C6—C1	119.12 (13)	N1—C13—C12	108.61 (14)
C5—C6—C7	119.03 (12)	N1—C13—H13	125.7
C1—C6—C7	121.83 (12)	C12—C13—H13	125.7
O1—C7—C8	121.73 (13)	C13—N1—C10	109.35 (12)
O1—C7—C6	119.52 (13)	C13—N1—H1N	125.1 (10)
C8—C7—C6	118.74 (12)	C10—N1—H1N	124.9 (10)
			
C6—C1—C2—C3	1.3 (2)	O1—C7—C8—C9	17.6 (2)
C1—C2—C3—C4	−0.4 (2)	C6—C7—C8—C9	−163.76 (13)
C2—C3—C4—C5	−1.2 (2)	C7—C8—C9—C10	−172.34 (13)
C3—C4—C5—C6	1.9 (2)	C8—C9—C10—N1	1.1 (2)
C4—C5—C6—C1	−1.0 (2)	C8—C9—C10—C11	173.37 (14)
C4—C5—C6—C7	−179.23 (12)	N1—C10—C11—C12	0.50 (16)
C2—C1—C6—C5	−0.7 (2)	C9—C10—C11—C12	−172.82 (13)
C2—C1—C6—C7	177.54 (13)	C10—C11—C12—C13	−0.54 (17)
C5—C6—C7—O1	22.6 (2)	C11—C12—C13—N1	0.37 (17)
C1—C6—C7—O1	−155.56 (14)	C12—C13—N1—C10	−0.06 (16)
C5—C6—C7—C8	−156.04 (13)	C11—C10—N1—C13	−0.27 (15)
C1—C6—C7—C8	25.76 (19)	C9—C10—N1—C13	173.41 (13)

### Hydrogen-bond geometry (Å, º)

Cg1 and Cg2 are the centroids of the (C1–C6) phenyl and (N1/C10–C13) 1H-pyrrole rings, respectively.

**Table:**

*D*—H···*A*	*D*—H	H···*A*	*D*···*A*	*D*—H···*A*
N1—H1*N*···O1^i^	0.872 (17)	2.119 (17)	2.9561 (18)	160.7 (14)
C2—H2···*Cg*1^ii^	0.93	2.82	3.450 (2)	126
C5—H5···*Cg*1^iii^	0.93	2.91	3.5233 (19)	124
C9—H9···*Cg*2^iv^	0.93	3.00	3.6207 (18)	126
C13—H13···*Cg*2^v^	0.93	2.95	3.593 (2)	127

Symmetry codes: (i) *x*−1, *y*, *z*; (ii) −*x*, −*y*, −*z*+1; (iii) −*x*+1, −*y*+1, −*z*+1; (iv) −*x*+1, −*y*, −*z*+1; (v) −*x*, −*y*+1, −*z*+1.
